# Efficient and lightweight long-range modeling for 3d point cloud classification and segmentation

**DOI:** 10.1371/journal.pone.0352793

**Published:** 2026-07-16

**Authors:** Dongzhen Liu, Yuzhong Deng, Jianxiao Zou, Shicai Fan

**Affiliations:** 1 School of Automation Engineering, University of Electronic Science and Technology of China, Chengdu, Sichaun, China; 2 Shenzhen Institute for Advanced Study, University of Electronic Science and Technology of China, Shenzhen, Guangdong, China; Leibniz University Hannover, GERMANY

## Abstract

3D point clouds, with their compact structural representation and rich geometric information, have become fundamental data sources for visual understanding tasks in computer vision, robotics, and intelligent systems.Despite extensive progress, many existing methods still place strong emphasis on local geometric modeling while exhibiting limited ability to capture global long-range contextual dependencies. Moreover, the increasing architectural complexity of modern models often leads to high computational cost and memory consumption. In this paper, we propose Point BiLSTM, an efficient and lightweight framework for 3D point cloud classification and segmentation. The core of the proposed method is a bidirectional long short-term memory (BiLSTM)-based sequencer module, which models long-range contextual dependencies among points with linear computational complexity, enabling effective global feature learning at a low cost. Considering the unordered nature of point clouds, we further propose a Mixed Sequence Soft Cross-Entropy Loss that jointly supervises fixed-order and randomly permuted point sequences during training. This design explicitly enhances robustness to permutation ambiguity and improves training stability. Extensive experiments conducted on three widely used benchmarks—ModelNet40, ScanObjectNN, and ShapeNet Part—demonstrate that Point BiLSTM achieves highly competitive performance. In particular, the proposed method attains the fastest inference speed on both idealized and real-world datasets, outperforming current state-of-the-art methods by 30.2% and 54.2%, respectively. In addition, Point BiLSTM significantly reduces computational complexity and memory consumption, providing an effective solution for efficient point cloud learning. Our code will be available at https://github.com/wendaodao04/PointBiLSTM.

## Introduction

3D point clouds provide a direct representation of the geometric structure of the real world and have been widely used in intelligent manufacturing [1], robotic manipulation [2] and 3D reconstruction [3]. Unlike regular grid-based data, point clouds describe three-dimensional space in a discrete, sparse, and unordered manner, which gives them inherent advantages in representing complex geometric structures. However, this unstructured nature also poses significant challenges for efficient and robust feature learning. With the rapid development of LiDAR and depth sensing technologies, the scale of point cloud data continues to grow. How to maintain high recognition accuracy while controlling computational complexity and memory consumption has become a critical issue for advancing point cloud understanding methods toward practical applications.Point cloud classification and segmentation are two fundamental tasks in point cloud understanding. Point cloud classification focuses on learning global semantic representations for an entire 3D object. In contrast, point cloud segmentation requires fine-grained semantic prediction for individual points. Compared with classification, segmentation generally requires more effective modeling of local geometric structures and global contextual relationships under irregular point distributions, making it a more challenging task.

Existing point cloud understanding approaches can be broadly categorized into methods based on regular representations [4–8] and methods that operate directly on point sets [9–20]. The former typically transform point clouds into regular structures, such as voxels [4–7], enabling the use of convolutional neural networks [21]. While these methods exhibit certain advantages in local geometric modeling, the voxelization process inevitably introduces spatial information loss, and their computational and memory costs increase rapidly with resolution, limiting their applicability in high-precision or resource-constrained scenarios. In contrast, direct point-based methods avoid intermediate representations and are more suitable for fine-grained geometric modeling. Representative examples include the PointNet [9–11] family based on multilayer perceptrons and subsequent approaches that incorporate local neighborhood aggregation. Although these methods have achieved substantial progress in classification and segmentation tasks, their modeling capability still largely relies on local feature stacking, with relatively limited ability to capture global contextual relationships.

To enhance global modeling capability, Transformer-based architectures and their variants [12,15,22–24] have recently been introduced into the point cloud domain. By leveraging self-attention mechanisms, these methods explicitly model long-range dependencies among points and have achieved strong performance on multiple benchmark datasets. Meanwhile, emerging sequence modeling frameworks such as state-space models [25,26] have also been explored for point cloud feature learning to improve efficiency. However, such approaches typically depend on complex operator designs or exhibit quadratic time and memory complexity [27], making them difficult to scale efficiently as the number of points increases. From a practical perspective, the usability of a model is determined not only by peak accuracy but also by inference speed, memory footprint, and overall computational cost. Consequently, how to preserve global modeling capability while constructing more efficient and scalable point cloud learning frameworks remains an open research problem.

From a data-structural perspective, a point cloud is inherently an unordered set of points. Although prior studies have attempted to impose sequential structures through spatial sorting or local aggregation [13,15,28,29], introducing any fixed ordering may lead to additional uncertainty and potential bias, which has long raised concerns about directly applying traditional sequence models to point cloud tasks. However, the strength of sequence models does not lie in their reliance on a specific “correct order,” but rather in their ability to efficiently model long-range dependencies. If a model can be guided to learn feature representations that are robust to order variations without depending on a particular point sequence, sequence modeling frameworks may offer a lightweight and effective alternative for global point cloud modeling.

Based on this observation, we propose Point BiLSTM, a long-range contextual modeling framework for 3D point cloud classification and segmentation. The proposed method adopts a bidirectional long short-term memory (BiLSTM) network as the core sequence modeling module and employs a residual structure to capture bidirectional dependencies among point features, enabling effective aggregation of global semantic information. To further mitigate the uncertainty introduced by point cloud unorderedness, we introduce a Mixed Sequence Soft Cross-Entropy Loss, which jointly supervises fixed-order and randomly permuted sequence views during training, encouraging the model to learn feature representations that are robust to variations in point ordering.The main contributions of this work are summarized as follows:

1. We propose a point cloud learning framework based on residual bidirectional long short-term memory networks, which captures long-range contextual dependencies with linear computational complexity and low memory overhead, making it suitable for both point cloud classification and segmentation tasks.
2. We introduce a mixed sequence soft cross-entropy loss that jointly supervises fixed and randomly permuted point sequences, effectively alleviating sensitivity to point ordering while improving training stability.
3. Experimental results on multiple standard point cloud benchmarks demonstrate that the proposed method achieves a favorable balance among classification accuracy, computational cost, and inference efficiency.

## Related work

### Methods based on convolution operators

Many existing approaches apply convolutional operations to 3D point clouds by designing specialized convolution kernels adapted to irregular point distributions [21,28,30–34]. Spherical CNN [32] addresses rotation variance by introducing spherical convolutions, while PointCNN [28] learns a permutation of unordered points to enable convolution-like operations. KPConv [21] defines convolution weights by locating kernel points in Euclidean space, and SpiderCNN [30] formulates continuous convolution kernels using parametric functions over local neighborhoods. InterpConv [33] further introduces interpolated convolution to capture geometric relationships between points and convolution weights, at the cost of expensive neighborhood search and sampling procedures.

Although these convolution-based methods have achieved strong performance on various point cloud understanding tasks, they generally rely on carefully customized kernel designs and complex local operators. Such designs make it difficult to construct a unified and efficient framework that generalizes well across different tasks, while often incurring non-negligible computational overhead.

### Methods based on the transformer

The Transformer architecture has achieved remarkable success in natural language processing and image understanding [35,36], which has motivated extensive research on applying attention mechanisms to 3D point cloud processing. A variety of Transformer-based models have been proposed for point cloud classification and segmentation [4,12,14–16,22,23,37]. Point Transformer [14] pioneers the use of self-attention for point clouds by introducing vector self-attention to aggregate local features, while PVT [4] combines sparse voxel attention with point-based relative attention to leverage both voxel and point representations. Fast Point Transformer [37] reduces spatial complexity by designing lightweight local attention, and Stratified Transformer [12] expands the receptive field through hierarchical sampling strategies.

Despite their strong performance, Transformer-based methods generally suffer from high computational and memory costs due to the quadratic complexity of self-attention. As the number of points increases, these costs become a significant bottleneck, limiting scalability and efficiency. This motivates the exploration of alternative mechanisms for capturing long-range dependencies with lower computational overhead.

### Methods based on LSTM

Long short-term memory (LSTM) networks and their variants have been widely used in sequence modeling tasks such as natural language processing [38], video analysis [39], and time series prediction [40]. By alleviating the vanishing gradient problem of recurrent neural networks, LSTM models are capable of capturing long-range dependencies in sequential data. In 3D vision, LSTM-based approaches have mainly been applied to temporally ordered point cloud streams. For example, LSTM networks have been used to model temporal information in multi-frame point cloud object detection [41], point cloud sequence prediction [42], and gesture recognition tasks [43]. These methods primarily focus on exploiting temporal continuity across consecutive point cloud frames. A small number of studies have explored LSTM-based architectures for static point cloud analysis [29], such as shape classification using fixed point sequences. Recent studies have further investigated point cloud semantic segmentation under few-shot and multimodal learning settings by leveraging cross-modal semantic priors and multimodal feature interaction [44–46]. These approaches improve semantic understanding capability under limited supervision conditions and reflect recent trends in generalized semantic learning for point cloud segmentation. However, existing approaches are generally limited to specific tasks and do not explicitly address permutation ambiguity or efficient long-range contextual modeling in unordered point sets. Consequently, the potential of classical sequence models for efficient and robust static point cloud understanding remains insufficiently explored.

### Proposed method

**Fig 1:**
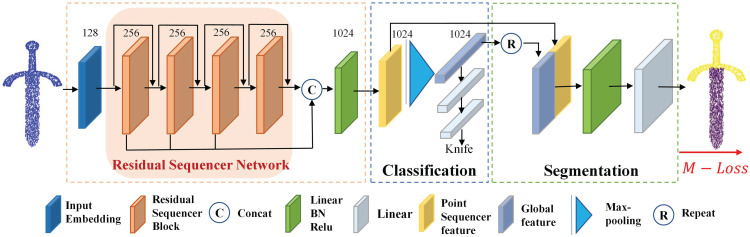
Point BiLSTM frameworks for 3D point classification and segmentation. It includes classification and segmentation networks. The specific details of the residual sequencer block are illustrated in Fig 2. “M−Loss” represents the loss function proposed in this paper.

Our proposed Point BiLSTM architecture is illustrated in Fig 1. It follows a hierarchical point processing pipeline, where standard sampling and neighborhood grouping operations are used to extract local structures. In the following sections, we describe each component in detail.

### Bidirectional LSTM

Bidirectional Long Short-Term Memory (BiLSTM) networks are a class of recurrent models designed to capture long-range dependencies in sequential data by jointly modeling forward and backward contextual information [47]. Unlike unidirectional recurrent models, BiLSTM processes an input sequence in both temporal directions, enabling each element to be informed by preceding and succeeding context simultaneously.

Given an input point feature sequence $x=(x_{1},x_{2},\ldots ,x_{T})$, a BiLSTM consists of a forward LSTM and a backward LSTM. The forward LSTM encodes contextual information in the original order, producing hidden states $\left\{{\vec{h}}_{t}\right\}$, while the backward LSTM processes the reversed sequence and produces hidden states $\left\{{\overset{\leftarrow }{h}}_{t}\right\}$. The output representation at each position is obtained by combining the hidden states from both directions:


$$\begin{array}{c} {𝐅}_{t}=\phi ({\vec{𝐡}}_{t},\;{\overset{\leftarrow }{𝐡}}_{t}), \end{array}$$ (1)

where ϕ(·) denotes a feature fusion operation, implemented as concatenation followed by a linear projection in our framework. The resulting representation ${𝐅}_{t}$ encodes bidirectional contextual information and serves as the output feature for the corresponding point.

In the context of static point cloud understanding, we treat point features as sequence elements without assuming a semantically meaningful order. The advantage of BiLSTM in this setting does not lie in exploiting a specific point ordering, but in its ability to efficiently propagate contextual information across the entire sequence with linear computational complexity. This property makes BiLSTM particularly suitable for modeling long-range dependencies in point clouds, providing an effective alternative to attention-based mechanisms with significantly lower computational and memory cost.

In our framework, the BiLSTM module functions as the core sequence modeling component and is integrated into a residual architecture, as described in the following subsection.

### Residual sequencer block

Although point clouds are inherently unordered sets, organizing point features into sequences provides an effective way to model long-range contextual dependencies. To explicitly incorporate spatial information into the sequence modeling process, we introduce a relative positional encoding term δ, which represents the relative positional relationship between neighboring points. This positional encoding is fused with the features learned by the BiLSTM to enhance spatial awareness during sequence modeling. Specifically, the positional encoding function and the proposed Sequencer Layer are defined as follows:


$$\begin{array}{c} \delta =\;\theta (x_{i}-x_{j}) \end{array}$$ (2)

$$\begin{array}{c} y_{i}=\sum_{x_{i}\in X(i)}\varphi \left(\gamma \left(x_{i}\right)\times \delta \right) \end{array}$$ (3)

**Fig 2:**
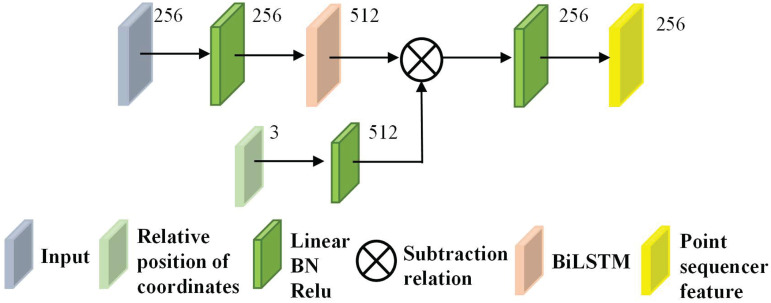
Residual Sequencer Block.

Here, $x_{i}$ and $x_{j}$ represent the 3D point coordinates for points *i* and *j*, respectively. The encoding function θ is an MLP consisting of two linear layers and one ReLU nonlinearity. x(i)⊆ X denotes a set of points in the local neighborhood of $x_{i}$ (K nearest neighbors). The mapping function φ is an MLP with one linear layer and a ReLU nonlinearity. γ represents the BiLSTM network. The positional information δ is directly fused with the feature information γ through a multiplicative relation, enhancing the spatial awareness of the feature representation. This approach allows the BiLSTM to not only capture the local context of each point but also combine the spatial information of the points, facilitating the capture of global spatial dependencies. The sequencer layer is illustrated in Fig 2.

By fusing sequential features with relative positional encodings, the proposed Sequencer Layer explicitly injects spatial awareness into the sequence modeling process. This design enables the BiLSTM to capture local geometric relationships while effectively modeling long-range contextual dependencies, thereby achieving global feature aggregation without relying on attention mechanisms. The overall structure of the residual sequencer block is illustrated in Fig 2 and highlighted in Fig 1. The block consists of a BiLSTM module, a linear projection layer, and a residual connection. The linear projection layer is employed to align the feature dimensions between the input and output of the sequencer, ensuring optimization stability and effective information propagation when stacking multiple layers.

### Mixed sequence soft cross-entropy loss

Soft cross-entropy loss is widely adopted in point cloud classification tasks. It can be regarded as a variant of the standard cross-entropy loss with the introduction of label smoothing [48]. The standard cross-entropy loss is defined as


$$\begin{array}{c} ℒ=-\sum_{i=1}^{K}p_{i}\log q_{i}, \end{array}$$ (4)

where *K* denotes the number of classes, $q_{i}$ represents the predicted probability for class *i*, and $p_{i}$ denotes the ground-truth distribution:


$$\begin{array}{c} p_{i}=\left\{ \begin{array}{cc} 1, & i=y,\\ 0, & i\neq y. \end{array}\right. \end{array}$$ (5)

To improve generalization ability and avoid overconfident predictions during training, label smoothing is introduced. The smoothed label distribution is defined as


$$\begin{array}{c} p_{i}=\left\{ \begin{array}{cc} 1-\varepsilon , & i=y,\\ \frac{\varepsilon }{K-1}, & i\neq y, \end{array}\right. \end{array}$$ (6)

where ε is a small hyperparameter controlling the smoothing strength.

However, point cloud data inherently exhibit an unordered structure, which introduces ambiguity when modeling them as sequences. To better exploit the complementary characteristics of ordered and unordered representations, we propose a mixed supervision strategy for sequence learning.

**Fig 3:**
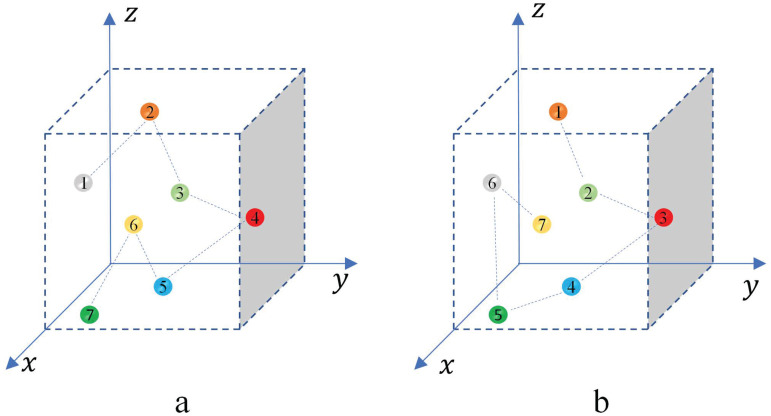
Ordered and unordered point cloud sequences. (a) A point cloud sequence with a randomly fixed order. (b) An arbitrarily permuted point cloud sequence with a different ordering from (a). Spheres with the same color represent the same point, and the numbers on the spheres indicate the point order in each sequence.

Specifically, two types of point cloud sequences are constructed. The first sequence is generated by fixing a random point order, which provides a consistent sequential view for feature extraction. The second sequence is produced through random permutations, which introduces diverse structural variations and enhances robustness. As illustrated in Fig 3, these two sequence views contain complementary geometric information that can benefit downstream tasks.

To jointly learn from these two views, we propose a mixed sequence soft cross-entropy loss defined as


$$\begin{array}{c} {ℒ}_{\text{M-Loss}}=\alpha {ℒ}_{i}+(1-\alpha ){ℒ}_{j}, \end{array}$$ (7)

where ${ℒ}_{i}$ represents the loss computed from the fixed-order point cloud sequence, while ${ℒ}_{j}$ corresponds to the loss obtained from the randomly permuted sequence. The parameter α∈[0,1] controls the trade-off between these two supervision signals.

By integrating both ordered and permutation-based supervision, the proposed M-Loss encourages the network to learn more robust and comprehensive representations from point cloud sequences.

### Experiments

In this section, we comprehensively evaluate the Point BiLSTM model on several benchmark datasets. Specifically, ModelNet40 [49] and ScanObjectNN [50] are used for shape classification, ShapeNet parts dataset [51] serves as the dataset for part segmentation, respectively.Subsequently, detailed ablation experiments demonstrate the effectiveness of Point BiLSTM in the process of point cloud feature learning.

### Datasets and implementation details

**Datasets.** We evaluate the proposed Point BiLSTM on three widely used benchmarks for 3D point cloud understanding, covering both classification and segmentation tasks.

ModelNet40 [49] is a synthetic CAD dataset consisting of 12,311 shapes from 40 object categories. Following the official split, 9,843 shapes are used for training and 2,468 for testing. This dataset is commonly adopted to evaluate the shape-level classification ability of point cloud models under clean and well-aligned conditions.

ScanObjectNN [50] is a real-world point cloud dataset captured by RGB-D sensors. It contains 15 object categories with 2,902 unique object instances and includes challenging factors such as background clutter, occlusion, and noise. In our experiments, we focus on the most challenging variant, PB_T50_RS, which introduces random background points and partial observations. ScanObjectNN is considered a strong benchmark for evaluating the robustness of point cloud classification methods.

ShapeNet Part [51] is a large-scale dataset for 3D part segmentation, containing 16 object categories and 50 annotated part labels. Each shape is associated with fine-grained per-point part annotations, making it a standard benchmark for evaluating point-level segmentation performance.

**Implementation details.** For all experiments, we uniformly sample a fixed number of points from each shape as network input. Specifically, 1,024 points are sampled for both ModelNet40 and ScanObjectNN, while 2,048 points are used for ShapeNet Part segmentation following common practice. The point coordinates are normalized to zero mean and unit sphere before being fed into the network. For classification tasks, stochastic gradient descent (SGD) with a momentum of 0.9 is employed for optimization. The initial learning rate follows the standard setting used in previous works and is decayed using a cosine annealing strategy. All classification models are trained for 300 epochs on ModelNet40 and 350 epochs on ScanObjectNN with a batch size of 32. For the part segmentation task, we follow the standard training protocol adopted in previous methods, including data augmentation operations such as random scaling and jittering, and train the model for 350 epochs. The proposed mixed sequence soft cross-entropy loss is applied to both classification and segmentation tasks. Unless otherwise specified, all hyperparameters are kept consistent across different datasets to ensure fair comparisons. In all experimental result tables, bold numbers indicate the best performance in each category, while the symbol “-” denotes that the corresponding metric is not reported in the original paper or official implementation. Test Speed represents the number of samples processed per second. All experiments are conducted on two Tesla P40 GPUs, and all reported test speeds are evaluated under the same hardware environment to ensure fair comparisons. The parameter counts and FLOPs reported in the comparison tables are obtained from the corresponding references.

### Results on ModelNet40

The classification results on ModelNet40 are summarized in Table 1. Point BiLSTM achieves an overall accuracy (OA) of 93.4%, which is competitive with recent state-of-the-art methods.Compared with PointMLP, our method yields slightly lower mean class accuracy (mAcc), but requires significantly fewer parameters (3.99 MB vs. 12.6 MB) and achieves substantially higher inference speed (574 vs. 154 samples/s). Compared with the previously fastest method, Multi-DL, the proposed Point BiLSTM achieves more than a 30% improvement in inference speed, while simultaneously attaining higher classification accuracy. This highlights the efficiency advantage of the proposed sequence-based modeling strategy.An important observation from Table 1 is that Point BiLSTM achieves a favorable balance between accuracy and computational efficiency.While several high-performing methods rely on deep MLP stacks or attention-based mechanisms, our approach attains comparable performance with a much lighter model. Notably, the inference speed of Point BiLSTM is approximately 3.7× faster than that of PointMLP, indicating that bidirectional sequence modeling enables effective long-range context aggregation without excessive architectural depth. Moreover, compared to attention-based models such as PCT and PointConT, Point BiLSTM avoids quadratic complexity with respect to the number of points, resulting in reduced FLOPs and faster execution. These results demonstrate that Point BiLSTM can effectively capture global shape semantics while maintaining high computational efficiency.

**Table 1:** Classification Results on the ModelNet40 Dataset.

Method	Input	mAcc (%)	OA (%)	Param. (MB)	Test Speed	FLOPs (G)
PointNet [9]	1024×3	86.2	89.2	3.5	–	0.5
PointNet++ [10]	1024×3	–	91.9	**1.5**	425	1.7
PointCNN [28]	1024×3	88.1	92.5	0.6	–	0.9
DGCNN [13]	1024×3	90.2	92.9	1.8	503	2.4
KPConv [21]	1024×3	–	92.9	–	–	–
PCT [15]	1024×3	–	93.2	2.88	325	2.17
PointMLP [11]	1024×3	91.4	**93.6**	12.6	154	15.73
PointConT (no voting) [22]	1024×3	–	92.8	14.66	143	2.59
TNPC [24]	1024×3	**91.6**	93.3	4.73	–	–
PointMamba [26]	1024×3	–	92.8	12.3	–	3.6
Mamba3D [25]	1024×3	–	93.4	16.9	43	3.25
Multi-DL [29]	1024×3	91.0	93.3	8.73	441	8.9
**Ours**	1024×3	90.8	93.4	3.99	**574**	2.46

### Results on ScanObjectNN

The experimental results on the ScanObjectNN dataset are reported in Table 2. Point BiLSTM achieves an mAcc of 86.7% and an OA of 87.9%, demonstrating competitive performance under challenging real-world conditions with background noise and occlusions. Although Mamba3D attains slightly higher overall accuracy, it involves a substantially larger model size (16.9 MB vs. 3.99 MB) and exhibits significantly lower inference speed (43 vs. 407 samples/s). In contrast, Point BiLSTM provides a more favorable trade-off among memory consumption, computational complexity, and accuracy, making it more suitable for scenarios with limited computational resources. Further observations from Table 2 indicate that Point BiLSTM exhibits a relatively small gap between mAcc and OA, suggesting more balanced performance across different object categories. This implies that the proposed bidirectional sequence modeling strategy is effective in capturing global contextual information even in the presence of background interference and partial observations.

**Table 2:** Classification Results on the ScanObjectNN Dataset.

Method	Input	mAcc (%)	OA (%)	Param. (MB)	Test Speed	FLOPs (G)
PointNet [9]	1024×3	63.4	68.2	3.5	–	0.5
PointNet++ [10]	1024×3	75.4	77.9	**1.5**	–	1.7
PointCNN [28]	1024×3	75.1	78.5	0.6	–	0.9
DGCNN [13]	1024×3	73.6	78.1	1.8	–	2.4
PCT [15]	1024×3	80.5	83.3	2.88	158	2.17
PointMLP [11]	1024×3	83.9	85.4	12.6	153	15.73
SPoTr [23]	1024×3	86.8	88.6	–	–	–
PointConT (no voting) [22]	1024×3	–	87.1	14.66	122	2.59
TNPC [24]	1024×3	79.8	81.4	4.73	–	–
PointMamba [26]	1024×3	–	87.9	12.3	–	3.6
Mamba3D [25]	1024×3	–	**88.2**	16.9	43	3.25
Multi-DL [29]	1024×3	85.5	87.0	8.73	264	8.9
**Ours**	1024×3	**86.7**	87.9	3.99	**407**	2.46

**Fig 4:**
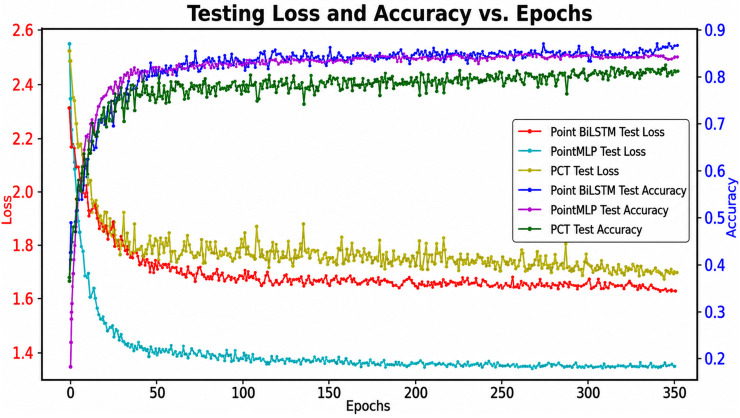
Comparison of Convergence Speed and Stability Among Different Models.

Fig 4 compares the training convergence behavior of different models. Point BiLSTM demonstrates stable convergence throughout the training process. Compared with PCT, the proposed method shows improved training stability, which can be attributed to the explicit modeling of point order variations introduced by the mixed sequence soft cross-entropy loss during training. Although PointMLP exhibits slightly smoother convergence curves, it relies on more complex nonlinear mapping structures, leading to higher training and inference costs. Overall, the experimental results on ScanObjectNN further verify that Point BiLSTM remains effective in realistic point cloud classification scenarios and exhibits strong robustness.

### Results on ShapeNet part

**Fig 5:**
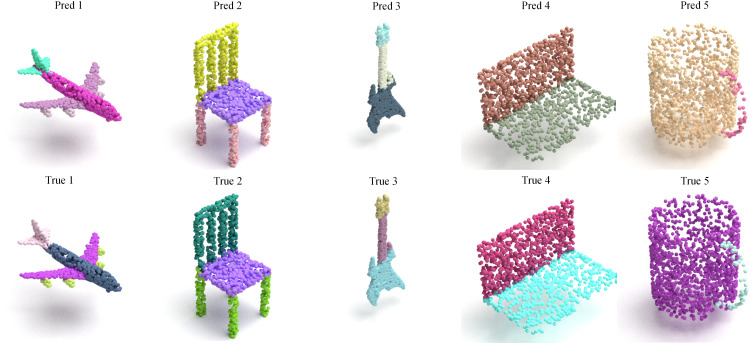
Visualization results for part segmentation using Point BiLSTM. Points aremarked with different colors based on the parts to which they belong. The first rowshows the model’s predicted results, and the second row displays the Ground Truth.

In addition to shape classification, we further evaluate the proposed Point BiLSTM framework on the part segmentation task, and the quantitative results are reported in Table 3. Point BiLSTM achieves an instance-level mIoU of 85.5%. Although the proposed framework is primarily designed for efficient global context modeling, it still demonstrates good generalization capability on the part segmentation task. The results indicate that bidirectional sequence modeling is effective in capturing structural consistency and long-range contextual dependencies, which are particularly important for part segmentation of objects with coherent global geometry. For objects with highly complex local structures or fine-grained part decompositions, methods that emphasize intensive local feature aggregation may achieve higher segmentation accuracy. This observation reflects a common trade-off between global context modeling and fine-grained local representation, and is consistent with the design philosophy of Point BiLSTM, which prioritizes efficiency and long-range dependency modeling rather than exhaustive local detail extraction. Compared with attention-based approaches, Point BiLSTM attains comparable instance-level segmentation performance while employing a considerably simpler network architecture. This suggests that explicit bidirectional sequence modeling can serve as a viable and efficient alternative for capturing global dependencies in part segmentation, without relying on computationally expensive self-attention mechanisms. In addition, Fig 5 presents qualitative visualizations of part segmentation results.

**Table 3:** Part Segmentation Results on the ShapeNet Part Dataset.

Method	Ins IoU	aero	bag	cap	car	chair	earph.	guitar	knife	lamp	laptop	m.bike	mug	pistol	rocket	skateb.	table
PointNet [9]	83.7	83.4	78.7	82.5	74.9	89.6	73.0	91.5	85.9	80.8	95.3	65.2	93.0	81.2	57.9	72.8	80.6
PointNet++ [10]	85.1	82.4	79.0	87.7	77.3	90.8	71.8	91.0	85.9	83.7	95.3	71.6	94.1	81.3	58.7	76.4	82.6
DGCNN [13]	85.2	84.0	83.4	86.7	77.8	90.6	74.7	91.2	87.5	82.8	95.7	66.3	94.9	81.1	63.5	74.5	82.6
PCNN [31]	85.1	82.4	**83.8**	88.2	80.5	91.1	76.2	91.9	87.6	84.9	95.8	70.7	95.3	82.4	63.6	72.7	82.8
P2Sequence [52]	85.2	82.6	81.8	87.5	77.3	90.8	77.1	91.1	86.9	83.9	95.7	70.8	94.6	79.3	58.1	75.2	82.8
SpiderCNN [30]	85.3	83.5	81.0	87.2	77.5	90.7	76.8	91.1	87.3	83.3	95.8	70.2	93.5	82.7	59.7	75.8	82.8
PCT [15]	**86.4**	**85.0**	82.4	89.0	**81.2**	**91.9**	71.5	91.3	88.1	**86.3**	95.8	64.6	95.8	83.6	62.2	77.6	83.7
PointMLP [11]	85.9	83.3	83.2	89.1	79.7	90.0	**80.3**	**91.8**	88.1	82.8	**96.2**	**76.9**	94.5	83.7	**66.3**	**82.2**	**83.5**
PointMamba [26]	85.8	–	–	–	–	–	–	–	–	–	–	–	–	–	–	–	–
Mamba3D [25]	85.7	–	–	–	–	–	–	–	–	–	–	–	–	–	–	–	–
**Ours**	85.5	83.6	80.7	**90.2**	79.2	89.9	73.8	91.7	**89.1**	80.4	**96.2**	70.3	**95.9**	**83.9**	61.4	**82.2**	**83.8**

### Ablation study

**Effect of network depth and sequence length.** We first investigate the influence of network depth by varying the number of residual sequencer blocks from 1 to 4, and the results are summarized in Table 4. When only a single block is used, the model exhibits limited representation capacity. Increasing the depth to two blocks leads to clear performance improvements. However, further increasing the depth to four blocks results in performance degradation, accompanied by increased computational and memory overhead. These results indicate that blindly increasing network depth does not necessarily benefit point cloud learning. A moderate depth achieves a better balance between representation capability, training stability, and efficiency. In all subsequent experiments, the number of residual sequencer blocks is set to two.

**Table 4:** Influence of Network Depth on Model Performance.

Network Depth	mAcc (%)	OA (%)
1	83.6	86.5
2	**86.7**	**87.9**
4	81.6	83.4

We further analyze the influence of sequence length on sequential contextual modeling performance, and the corresponding results are presented in Table 5. When the sequence length is relatively short, the model is unable to sufficiently capture long-range contextual dependencies, resulting in limited feature interaction capability. As the sequence length increases from 64 to 256, the classification performance consistently improves, demonstrating that richer contextual interaction contributes to more discriminative global representation learning. However, further increasing the sequence length to 512 yields only marginal performance gains while significantly increasing computational and memory costs. Moreover, excessively long sequences may propagate redundant or noisy contextual information during recurrent interaction, which can negatively affect model robustness. Therefore, an appropriate sequence length achieves a better trade-off between contextual modeling capability and computational efficiency. According to the experimental results, the sequence length is set to 256 in the final model configuration.

**Table 5:** Influence of sequence length on Model Performance.

Sequence Length	mAcc (%)	OA (%)
64	84.4	86.0
128	85.7	87.1
256	**86.7**	**87.9**
512	86.5	**87.9**

We also investigate the influence of different sampling strategies on sequence token construction and contextual interaction quality. The corresponding results are summarized in Table 6. Random Sampling exhibits relatively unstable performance due to irregular spatial point distributions, which may weaken sequential contextual interaction and reduce feature consistency. Uniform Sampling improves spatial coverage to some extent and achieves better performance than Random Sampling. In contrast, the proposed framework achieves the best performance when using FPS, since FPS preserves more uniformly distributed and representative geometric structures, thereby facilitating more stable long-range contextual dependency modeling. These results further demonstrate that representative spatial point distributions are essential for effective sequential feature learning in point cloud analysis.

**Table 6:** Influence of Sampling Strategy on Model Performance.

Sampling Strategy	mAcc (%)	OA (%)
Random Sampling	85.2	86.9
Uniform Sampling	85.7	87.3
Furthest Point Sampling	**86.7**	**87.9**

**Effectiveness of mixed sequence soft cross-entropy loss.** To evaluate the effectiveness of the proposed mixed sequence soft cross-entropy loss and investigate the sensitivity to the mixing coefficient α, we vary α from 0 to 1 with an interval of 0.25. The corresponding results are summarized in Table 7. Here, α=0 indicates that supervision is performed using only randomly permuted sequences, whereas α=1 corresponds to supervision using only fixed-order sequences. Intermediate values of α represent different mixing ratios between fixed-order and randomly permuted sequence supervision. As shown in Table 7, relying solely on a single sequence view leads to inferior performance compared with mixed supervision. In particular, when α=0, the model achieves the lowest performance, suggesting that supervision based only on randomly permuted sequences weakens the stability of sequential contextual learning. As α gradually increases from 0 to 0.5, both mAcc and OA consistently improve, indicating that introducing fixed-order sequence supervision provides more stable and discriminative contextual representations. However, further increasing α from 0.5 to 1.0 results in performance degradation, implying that excessive dependence on fixed-order sequences may reduce robustness to point-order variations. Overall, the best performance is achieved when α=0.5, demonstrating that a balanced combination of fixed-order and randomly permuted sequence supervision provides the most effective trade-off between order consistency and permutation robustness. These results further validate both the effectiveness of the proposed mixed sequence soft cross-entropy loss and the importance of selecting an appropriate mixing coefficient.

**Table 7:** Ablation Study of Different Loss Functions.

Loss Function	mAcc (%)	OA (%)
soft cross-entropy loss (α=0)	84.9	86.1
mixed sequence soft cross-entropy loss (α=0.25)	86.0	87.5
mixed sequence soft cross-entropy loss (α=0.5)	**86.7**	**87.9**
mixed sequence soft cross-entropy loss (α=0.75)	85.3	86.8
soft cross-entropy loss (α=1)	85.8	87.2

**Effectiveness of residual sequencer block.** We further investigate the contribution of the residual sequencer block and different sequence modeling units. Under the same network depth and loss function settings, three variants are compared: without sequence modeling, with LSTM, and with BiLSTM. The corresponding results are presented in Table 8. Removing the sequencer block leads to a significant performance degradation, indicating that sequential contextual interaction plays a critical role in point cloud representation learning. Introducing LSTM substantially improves performance by enabling long-range contextual dependency modeling. Furthermore, replacing LSTM with BiLSTM consistently achieves better results, demonstrating that bidirectional sequence modeling is more effective than unidirectional modeling for capturing contextual relationships in unordered point cloud data. By aggregating contextual information from both forward and backward directions simultaneously, BiLSTM provides more comprehensive sequential feature interaction and stronger global representation capability. We additionally analyze the effectiveness of incorporating relative positional encoding into the residual sequencer block, and the corresponding results are summarized in Table 9. Without relative positional encoding, the model performance decreases noticeably, indicating that sequential feature interaction alone is insufficient for fully capturing geometric relationships in point clouds. After introducing relative positional encoding, both mAcc and OA are consistently improved, demonstrating that explicit geometric priors enhance the spatial awareness and discriminative capability of sequence-based contextual modeling.

**Table 8:** Ablation Study of LSTM and BiLSTM Components.

LSTM	BiLSTM	mAcc (%)	OA (%)
×	×	78.8	81.5
✓	×	85.4	86.8
×	✓	**86.7**	**87.9**

**Table 9:** Ablation Study of relative positional encoding.

Setting	mAcc (%)	OA (%)
relative positional encoding (w/o)	85.6	87.1
relative positional encoding (w)	**86.7**	**87.9**

**Effectiveness of a randomly assigned but fixed point cloud order.** Finally, we study whether imposing a specific deterministic order benefits model performance. We compare randomly fixed orders with orders obtained by sorting points along the x-, y-, and z-axes. The results are reported in Table 10. All ordering strategies achieve comparable performance, with no consistent advantage observed for axis-based sorting. Moreover, deterministic sorting introduces additional preprocessing cost. These results indicate that the proposed framework does not rely on a specific point order and is capable of learning order-invariant representations through sequence modeling and mixed-sequence supervision.

**Table 10:** Influence of Different Sorting Methods on Model Performance.

Sorting Method	mAcc (%)	OA (%)
x-axis	86.68	87.89
y-axis	86.71	**87.94**
z-axis	86.69	87.90
Random	**86.73**	87.93

### Failure case analysis

**Fig 6:**
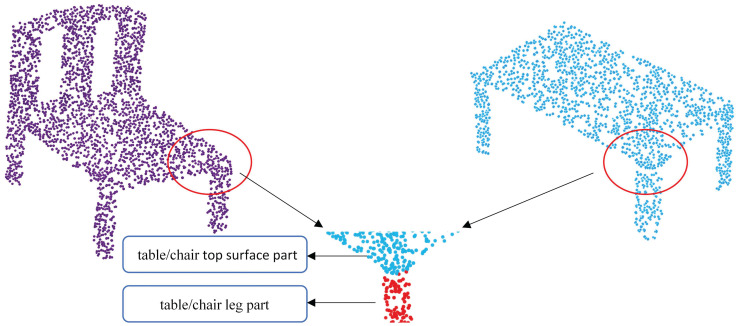
Visualization of representative failure cases. Geometrically similar local structures, such as the junction regions between table/chair legs and flat surface structures, may introduce contextual ambiguity during sequence-based feature interaction, leading to feature confusion. Different colors are used to facilitate distinguishing between the legs and flat surface structures of tables and chairs.

Although our method achieves competitive results on multiple public datasets, it still has certain limitations. As shown in Fig 6, tables and chairs contain highly similar structural components, particularly at the junctions between supporting legs and flat surfaces. These locally similar geometric structures may be misinterpreted as belonging to the same semantic category during sequence-based contextual modeling, leading to contextual feature ambiguity. While modeling long-range dependencies enhances global feature aggregation and cross-region contextual interaction, the model may still struggle to distinguish subtle differences in highly symmetric or repetitive local geometries, which can affect fine-grained semantic boundary discrimination. This highlights an inherent challenge of sequence-based modeling: local geometric similarity can conflict with global contextual information, causing similar local structures to be incorrectly aggregated into the same contextual representation and resulting in feature confusion. Future work may address these limitations by incorporating more discriminative geometry-aware contextual interaction mechanisms and adaptive sequence construction strategies to further improve the robustness of sequence-based point cloud understanding.

## Conclusion

In this paper, we presented Point BiLSTM, an efficient framework for 3D point cloud classification and segmentation based on bidirectional sequence modeling. By introducing a residual BiLSTM-based sequencer, the proposed method is able to capture long-range contextual dependencies with linear computational complexity, providing an effective mechanism for global feature aggregation. To address the unordered nature of point clouds, we further proposed a mixed sequence soft cross-entropy loss, which jointly supervises fixed-order and randomly permuted point sequences. This design explicitly encourages robustness to point order variations and improves training stability. Extensive experiments and ablation studies on multiple benchmark datasets demonstrate that Point BiLSTM achieves competitive performance while significantly reducing model complexity, memory usage, and inference latency. These results highlight that explicit bidirectional sequence modeling can serve as a lightweight and effective alternative for global context learning in point cloud understanding. Rather than replacing existing paradigms, this work provides a complementary perspective on point cloud modeling, bridging classical sequence learning and modern 3D perception. Future work will explore extending the proposed framework to other 3D tasks, such as object detection and scene-level understanding.
